# Aridity drives the response of soil total and particulate organic carbon to drought in temperate grasslands and shrublands

**DOI:** 10.1126/sciadv.adq2654

**Published:** 2024-10-04

**Authors:** Baoku Shi, Manuel Delgado-Baquerizo, Alan K. Knapp, Melinda D. Smith, Sasha Reed, Brooke Osborne, Yolima Carrillo, Fernando T. Maestre, Yu Zhu, Anping Chen, Kate Wilkins, Martin C. Holdrege, Andrew Kulmatiski, Catherine Picon-Cochard, Christiane Roscher, Sally Power, Kerry M. Byrne, Amber C. Churchill, Anke Jentsch, Hugh A. L. Henry, Karen H. Beard, Max A. Schuchardt, Nico Eisenhauer, Rafael Otfinowski, Yann Hautier, Huitao Shen, Yonghui Wang, Zhongwu Wang, Chengliang Wang, Daniela Francis Cusack, Alessandro Petraglia, Michele Carbognani, T’ai G.W. Forte, Luke Flory, Pengli Hou, Tao Zhang, Weifeng Gao, Wei Sun

**Affiliations:** ^1^ Institute of Grassland Science, Key Laboratory of Vegetation Ecology of the Ministry of Education, Jilin Songnen Grassland Ecosystem National Observation and Research Station, Northeast Normal University, Changchun 130024, China.; ^2^ Laboratorio de Biodiversidad y Funcionamiento Ecosistémico, Instituto de Recursos Naturales y Agrobiología de Sevilla (IRNAS), CSIC, Av. Reina Mercedes 10, Sevilla E-41012, Spain.; ^3^ Unidad Asociada CSIC-UPO (BioFun), Universidad Pablo de Olavide, Sevilla 41013, Spain.; ^4^ Department of Biology and Graduate Degree Program in Ecology, Colorado State University, Fort Collins, CO 80523, USA.; ^5^ Southwest Biological Science Center, US Geological Survey, Moab, UT 84532, USA.; ^6^ Department of Environment and Society, Utah State University, Moab, UT 84532, USA.; ^7^ Hawkesbury Institute for the Environment, Western Sydney University, Penrith, NSW 2751, Australia.; ^8^ Environmental Sciences and Engineering, Biological and Environmental Science and Engineering Division, King Abdullah University of Science and Technology, Thuwal 23955, Kingdom of Saudi Arabia.; ^9^ College of Life Science and Technology, Central South University of Forestry and Technology, Changsha 410004, China.; ^10^ Denver Zoological Foundation, Denver, CO 80205, USA.; ^11^ Department of Wildland Resources and the Ecology Center, Utah State University, Logan, UT 84322-5230, USA.; ^12^ Université Clermont Auvergne, INRAE, VetAgro Sup, UREP, Clermont-Ferrand 63000, France.; ^13^ Helmholtz Centre for Environmental Research, UFZ, Department Physiological Diversity, Leipzig 04318, Germany.; ^14^ German Centre for Integrative Biodiversity Research (iDiv) Halle-Jena-Leipzig. Puschstrasse 4, Leipzig, 04103, Germany.; ^15^ Department of Environmental Science and Management, California State Polytechnic University, Humboldt, Arcata, CA 95521, USA.; ^16^ Department of Ecology, Evolution and Behavior, University of Minnesota, St. Paul, MN 55108, USA.; ^17^ Department of Disturbance Ecology and Vegetation Dynamics, Bayreuth Center of Ecology and Environmental Research, University of Bayreuth, Universitätsstraße 30, Bayreuth 95447, Germany.; ^18^ Department of Biology, University of Western Ontario, London, ON N6A 5B7, Canada.; ^19^ Institute of Biology, Leipzig University, Puschstrasse 4, Leipzig 04103, Germany.; ^20^ Department of Biology, The University of Winnipeg, Winnipeg, MB R3B 2E9, Canada.; ^21^ Ecology and Biodiversity Group, Department of Biology, Utrecht University, Padualaan 8, Utrecht 3584 CH, Netherlands.; ^22^ Hebei Engineering Research Center for Geographic Information Application, Institute of Geographical Sciences, Hebei Academy of Sciences, Shijiazhuang 050021, China.; ^23^ Ministry of Education Key Laboratory of Ecology and Resource Use of the Mongolian Plateau & Inner Mongolia Key Laboratory of Grassland Ecology, School of Ecology and Environment, Inner Mongolia University, Hohhot 010021, China.; ^24^ College of Grassland, Resources and Environment, Inner Mongolia Agricultural University, Hohhot 010010, China.; ^25^ Department of Ecosystem Science and Sustainability, Colorado State University, Fort Collins, CO 80523, USA.; ^26^ Smithsonian Tropical Research Institute, Apartado, Balboa 0843-03092, Panama.; ^27^ Department of Chemistry, Life Sciences and Environmental Sustainability, University of Parma, Parma 43124, Italy.; ^28^ Agronomy Department, University of Florida, Gainesville, FL 32601, USA.; ^29^ State Environmental Protection Key Laboratory of Wetland Ecology and Vegetation Restoration, Northeast Normal University, Changchun, Jilin 130024, China.

## Abstract

The increasing prevalence of drought events in grasslands and shrublands worldwide potentially has impacts on soil organic carbon (SOC). We leveraged the International Drought Experiment to study how SOC, including particulate organic carbon (POC) and mineral-associated organic carbon (MAOC) concentrations, responds to extreme drought treatments (1-in-100-year) for 1 to 5 years at 19 sites worldwide. In more mesic areas (aridity index > 0.65), SOC and POC concentrations decreased by 7.9% (±3.9) and 15.9% (±6.2) with drought, respectively, but there were no impacts on MAOC concentrations. However, drought had no impact on SOC, POC, or MAOC concentrations in drylands (aridity index < 0.65). The response of SOC to drought varied along an aridity gradient, concomitant with interannual precipitation variability and standing SOC concentration gradients. These findings highlight the differing response magnitudes of POC and MAOC concentrations to drought and the key regulating role of aridity.

## Teaser

Drought reduces soil carbon in wetter regions, but drylands remain stable, showing aridity’s key role in carbon dynamics.

## INTRODUCTION

Soil organic carbon (SOC) is the largest terrestrial carbon (C) pool (*1*, *2*), and even small changes in SOC can have large effects on Earth’s climate. Drought, defined as a sustained, extended deficiency in precipitation, can be detected using standardized indices or long-term climate records (*3*, *4*). The frequency and duration of drought events in grasslands and shrublands are expected to increase with climate change, potentially having impacts on ecosystem C cycling (*5*–*7*). While there have been numerous studies on drought impacts on aboveground biomass and productivity around the world (*8*, *9*), our understanding of how drought influences SOC is quite limited.

Previous studies have shown that drought has variable effects on SOC, including increased, decreased, or unchanged SOC (*10*–*14*). For instance, SOC stocks were not notably affected by drought in seminatural grasslands and constructed old-field ecosystems (*10*, *14*). In contrast, soil C concentrations increased during the dry summer in a California annual grassland (*15*). A recent meta-analysis reported a 3.3% reduction in soil organic C content, primarily due to an 8.7% decrease in plant litter input and a 13.0% reduction in litter decomposition over a 1- to 13-year drought period (*13*). Three main reasons explain the limited understanding of how drought influences SOC. First, the responses of soil C pools and fluxes to drought are often synthesized across ecosystems using meta-analysis (*11*–*13*) rather than through standardized cross-site experiments. Interpreting the mechanisms and controls of SOC from meta-analyses can be complicated by variations in methods, including large differences in the nature and intensity of drought treatments (e.g., chronic rainfall reduction versus pulsed drought years). Second, previous works have generally treated SOC as a single entity without distinguishing particulate organic C (POC) and mineral-associated organic C (MAOC), which have very different origins and sensitivities to global change (*16*–*18*). Specifically, POC originates directly from the structural components of plants (*19*, *20*) and is more readily available to microbes. Drought reduces plant productivity and hence the input of plant products (above- and belowground litter) (*8*), which could cause subsequent decreases in POC. In contrast, MAOC forms through the mineral adsorption of dissolved organic C and microbial necromass, representing a longer-term, more stable fraction of SOC in soils than POC (*21*–*23*). Because of fundamental differences between POC and MAOC in their formation pathways and persistence mechanisms (*24*), they are predicted to respond differently to drought. Previous studies have shown that POC responds more quickly to other global change drivers as well, including warming, elevated CO_2_, N fertilization, and increased precipitation in the short-term compared to MAOC (*17*, *25*). Despite the importance of understanding the controls and consequences of drought for assessing and forecasting these C pools, empirical evidence comparing drought responses of POC and MAOC is limited. Last, most studies on climate change impacts on SOC fractions are conducted at a single site, limiting our capacity to understand and predict how environmental conditions regulate the response of ecosystem C cycling to drought across the globe. The effects of drought on SOC, POC, and MAOC depend on local climatic conditions, including mean annual precipitation (MAP) and interannual variations in precipitation. This is because these factors control all processes from C input from plant productivity to microbial respiration and ultimate loss from the ecosystem. In addition, edaphic factors such as standing SOC concentration can also drive responses, as C-rich soils can show particularly large C losses after intense droughts (*26*). Water-holding capacity (WHC) can be indicative of both soil texture and soil organic matter. As soil texture becomes finer, the available moisture storage generally increases, progressing from sands to loams and silt loams (*27*). Therefore, plants growing in coarse-textured soils with low WHC may be more susceptible to drought stress compared to those growing in silt loam soils with high WHC. Aridity level, as an integrative variable defined by the aridity index (AI) (the ratio of MAP to potential evapotranspiration), is expected to be of high importance. For example, plant and microbial growth in drylands (with low MAP and AI) tends to be more sensitive to drought than in mesic systems (*28*). However, ecosystems with low MAP and AI are expected to experience high historical climatic variability, so that plant and microbial growth could also be adapted to large interannual variations in precipitation (*29*). A global investigation across wide climatic gradients could provide powerful, scalable insights into the dynamics that dictate soil C responses to drought under varied environmental conditions.

**Fig. 1.**
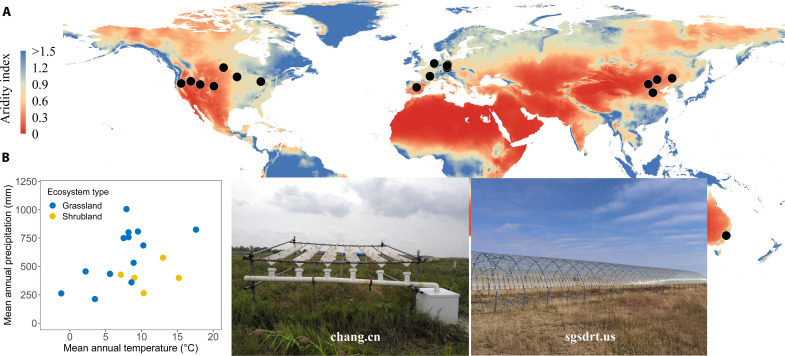
A globally distributed network of experiments to investigate the impacts of drought on soil carbon. (**A**) Experimental sites are displayed as black dots over a global AI map. The more arid an ecosystem is, the lower its AI. (**B**) Range of mean annual temperature and MAP of the 19 study sites, which are color-coded according to ecosystem type. Examples of drought shelters at representative sites are shown in the photos.

Here, we leveraged a network of standardized field experiments at diverse sites around the world [the International Drought Experiment (IDE)] to examine how extreme drought influenced SOC, POC, and MAOC concentrations in grasslands and shrublands. We imposed a historically extreme, 1-in-100-year drought treatment for 1 to 5 years at 19 globally distributed sites, encompassing a wide range of temperature and precipitation conditions (e.g., spanning more than 18.8°C in mean annual temperature and 793 mm in MAP) (Fig. 1 and table S1). Most sites (16 of 19) experienced drought conditions for 4 to 5 years (fig. S1). By using standardized drought and data collection methods, the IDE network allowed us to assess both the mechanisms and outcomes of drought and to provide a more robust estimate of the effects of drought across numerous scales (*30*, *31*). We hypothesized that, globally, (i) the response of SOC to drought would depend on the long-term aridity of a site, which can be measured with the AI and would differ among dryland and mesic systems because drought sensitivity of the control factors (e.g., plant and microbial growth and respiration) in soil C cycling varies with AI (*28*, *29*), and (ii) POC could be more sensitive to drought than MAOC because POC consists of labile fractions with shorter turnover times, which are easily consumed by microorganisms and rapidly decomposable, while MAOC bonds to mineral surfaces and is biochemically recalcitrant (*17*, *20*).

## RESULTS AND DISCUSSION

### Drought impacts on carbon concentrations

**Fig. 2.**
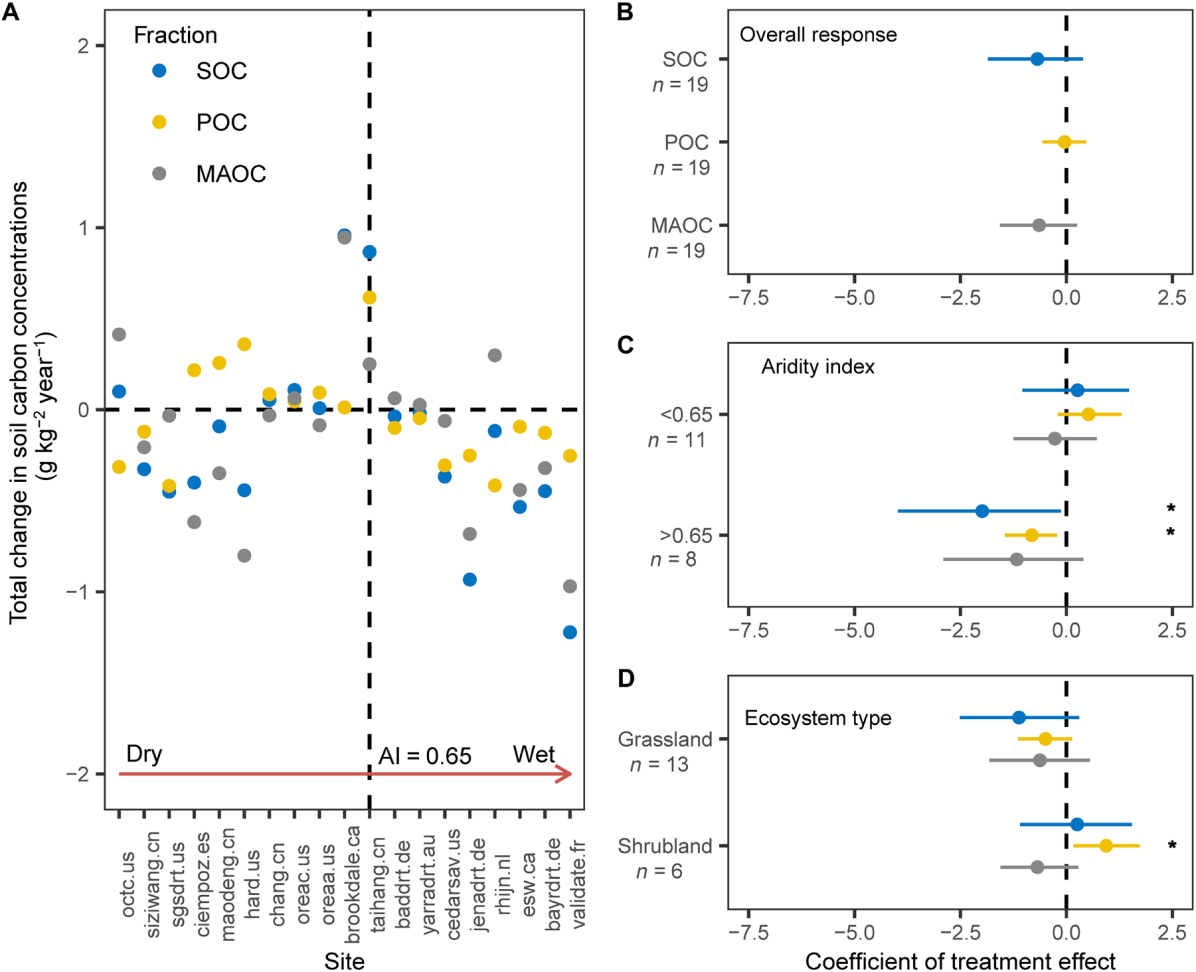
The responses of SOC, POC, and MAOC concentrations to experimental drought. (**A**) Total changes in SOC, POC, and MAOC concentrations across sites assembled by levels of AI. Water availability increases with higher AI. (**B**) Responses of SOC, POC, and MAOC concentrations to drought across all sites and as moderated by (**C**) AI and (**D**) ecosystem type. Points indicate mean treatment effects across all sites, error bars indicate 95% confidence intervals, and *n* indicates number of observations, *P < 0.05. Site codes are listed in table S1.

**Fig. 3.**
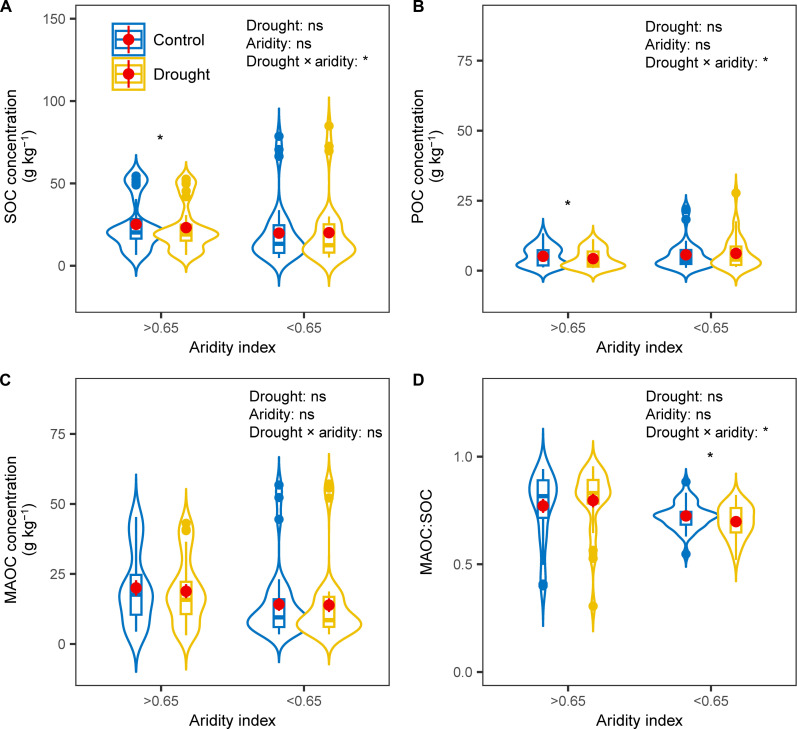
AI-moderated responses of soil carbon fractions to drought. The responses of (**A**) SOC, (**B**) POC, and (**C**) MAOC concentrations and (**D**) ratio between MAOC and SOC to drought in drylands (AI < 0.65) and mesic (AI > 0.65) ecosystems. Red dot shows the mean. *P < 0.05; ns, *P* > 0.05.

Experimentally imposed drought reduced SOC concentration by 3.1% (±2.6) across the 19 sites, but this effect was not statistically significant (*P* > 0.05; Figs. 2 and 3). This estimate aligns with a recent meta-analysis of 1344 measurements from 128 global manipulative studies, which reported a minor and statistically insignificant decrease in soil organic C pool under drought conditions (−4%; 95% confidence interval, −8 to −1%) (*12*). There were no overall notable effects of drought on POC and MAOC concentrations (*P* = 0.88 and *P* = 0.17).

Further analyses revealed that the responses of SOC concentrations to drought depended on ecosystem type (Fig. 2). In grasslands, drought trended toward overall negative effects on SOC, POC, or MAOC concentrations (*P* = 0.14, *P* = 0.15, and *P* = 0.32, respectively). In shrublands, drought increased POC concentration by 0.94 ± 0.39 g kg^−1^ year^−1^ (*P* < 0.05) and had no impact on SOC and MAOC concentrations (*P* = 0.71 and *P* = 0.17). Deng *et al.* (*13*) also found that in shrublands, SOC concentrations slightly increased in response to drought. This could be attributed to the fact that drought inhibited soil C decomposition more than it reduced soil C input, leading to a minor increase in the soil C pool under drought conditions (*11*, *13*).

Our analyses revealed that aridity largely regulated the responses of SOC to drought across a diversity of globally distributed sites. We used an AI value of 0.65 as a cutoff because it is widely used to represent the threshold between drylands and mesic ecosystems (*32*). In mesic areas (AI > 0.65), SOC and POC concentrations decreased by 7.9% (±3.9) and 15.9% (±6.2) with drought, respectively (all *P* < 0.05) (Figs. 2 and 3), while MAOC was not affected (*P* = 0.18). In contrast, drought had no impact on SOC, POC, or MAOC concentrations in drylands (AI < 0.65). Our results suggest that in dryland regions, where SOC concentrations are relatively low, increases in drought will have a limited impact on soil C concentrations. However, in mesic systems, increases in drought will significantly reduce soil organic C concentrations, potentially affecting key ecosystem services such as soil fertility, biodiversity, and climate regulation. Our study demonstrates that drought is particularly critical for SOC and POC in mesic ecosystems, where SOC concentrations, although initially higher than in arid and semiarid ecosystems, decrease with drought. These results also indicate that POC was more sensitive to drought than MAOC in mesic ecosystems, confirming our hypotheses. POC originates from particulate organic residues of plant, which are affected by plant productivity and are particularly vulnerable to climate change (*19*). In contrast, MAOC adheres to mineral surfaces and is relatively stable (*23*). Drought could induce a decrease in aboveground net primary productivity (ANPP; fig. S2), further decreasing POC concentrations. While particulate organic matter (POM) can be occluded within large aggregates, the rate at which POM is cycled within these aggregates is only slightly longer than that of free POM and is less pronounced than the turnover rate of organic matter associated with minerals (MAOM) (*33*). Our results highlight the importance of measuring POC and MAOC to advance our mechanistic understanding of how and why drought events affect SOC across global environmental gradients.

### Factors influencing SOC responses to drought

Drought reduced ANPP by 20.0% (±4.0) across the 19 sites (*P* < 0.05) (fig. S2). Drought trended toward overall negative effects on soil total N concentration, while it trended toward overall positive effects on soil phenols (*P* = 0.09 and *P* = 0.08). There were significant differences in ANPP, soil total N concentration, and microbial necromass C between the dryland and mesic systems (all *P* < 0.05). The WHC in dryland systems was, on average, 23.5% (±12.7) lower than that of in mesic systems.

**Fig. 4.**
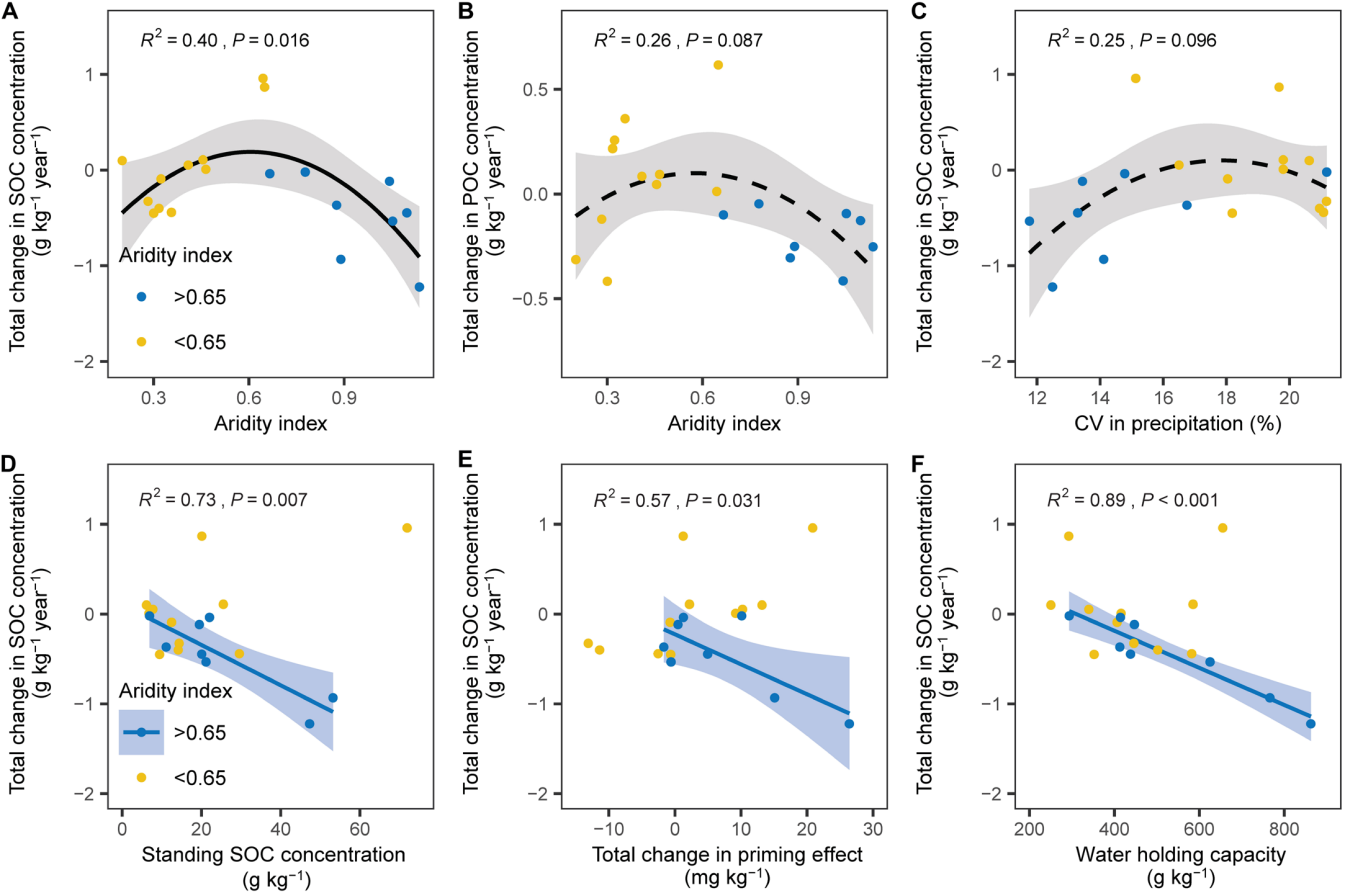
Environmental factors influencing SOC concentrations responses to drought. Relationships between (**A**) total changes in SOC concentrations and the AI, (**B**) total changes in POC concentrations and the AI, (**C**) total changes in SOC concentrations and the CV in precipitation, (**D**) standing SOC concentration, (**E**) total change in priming effect, and (**F**) water holding capacity. Solid lines indicate significant correlations, while dashed lines indicate nonsignificant correlations. Shaded areas reflect the 95% confidence interval for the line of fit.

We found that the relationship between absolute differences in total SOC and POC concentrations (ΔDDSOC and ΔDDPOC) and AI was best described by a second-order polynomial regression with thresholds at AI = 0.61 and 0.58, respectively (Fig. 4). The threshold for SOC (AI = 0.61) is close to the transition threshold (AI = 0.65) between dryland and mesic systems. These findings further support the idea that the response of SOC concentration to drought varies with AI and its pattern largely depends on the response of POC. Several mechanisms could explain this result. Net changes in SOC primarily reflect the balance between plant C input and soil organic matter decomposition (*34*, *35*). First, there were no notable effects of drought on soil C concentrations in arid and semiarid ecosystems with low AI and high coefficient of variation (CV) in precipitation (Fig. 4 and fig. S3). This could be because controlling factors (e.g., plant and microbial growth and respiration) for soil C turnover are adapted to high interannual precipitation variability (*29*). We found that the ANPP loss decreased with increasing interannual precipitation variability, which supports the above inference (fig. S3). Second, our results indicated that drought induced significant decreases in total SOC concentrations occurred in regions with high AI accompanied by high initial SOC concentration (Fig. 4 and fig. S3). This can be attributed to the initial SOC providing a substantial substrate for microbial activity (*26*), leading to increased CO_2_ release after drought events due to high substrate availability. Furthermore, our research also showed that in C-rich soils, fresh C inputs can result in a pronounced priming effect, leading to greater C losses (fig. S3). Consequently, the increase in priming effects induced by drought can reduce total SOC concentrations (Fig. 4). Last, we observed that WHC increased with the AI, and WHC in mesic systems was negatively correlated with the absolute differences in soil C concentrations (ΔDDSOC) across the studied sites (Fig. 4 and fig. S3). There were no significant correlations between the absolute differences or response ratios in soil C concentrations and changes in ANPP (fig. S4). This finding contradicts our expectation that plants in coarse-textured soils with low WHC would be more susceptible to drought stress (*27*), thereby reducing C inputs and soil organic C. The likely reason is that concurrent changes in soil organic matter decomposition mediate SOC responses. In mesic areas with a high AI, soils are often saturated or nearly saturated with water, whereas the optimal moisture content for microbial activity is approximately 60% of WHC (*36*, *37*). Thus, drought may enhance the decomposition of soil organic matter in these areas. Our results showed that drought increased microbial necromass C by 27.8% (±18.6) in mesic areas (fig. S2), indicating high microbial turnover or growth rates under drought conditions. This increase in microbial activity can stimulate soil organic matter decomposition, partly confirming our hypothesis.

Aridity is a primary driver of ecosystem structure and functioning at a global scale (*38*). It mediates the relationships between biodiversity and soil multifunctionality, as well as biodiversity and ecosystem stability across the globe (*39*, *40*). Our findings revealed that the response of SOC to drought varied along an aridity gradient, concomitant with CV in precipitation and standing SOC concentration gradients. These results highlight the fundamental role of environmental conditions, such as aridity, in explaining the responses of key ecosystem attributes, specifically SOC, to climate change, suggesting a more significant influence than previously reported (*11*, *13*, *38*).

There are several potential limitations to this study. On the one hand, although we used passive rainout shelters designed to mimic site-specific 100-year drought conditions, the severity of drought treatments may have differed across sites because of variability in ambient precipitation (*41*). That is, the actualized reduction in precipitation amounts at each site varied with the amount of annual precipitation received during the year(s) of drought treatment. However, 16 of our 19 sites experienced drought conditions for 4 to 5 years. This may have reduced the impact of variability in interannual precipitation on drought intensity. On the other hand, the nature and strength of SOC, POC, and MAOC responses to drought varied widely across sites, ranging from net losses to net gains (Fig. 2). While more research on a global scale is needed to validate our models, the response patterns of SOC, POC, and MAOC concentrations to drought across a wide range of grasslands and shrubland ecosystems provide valuable information that improves our capacity to predict the potential changes in soil C stocks under climate change.

In summary, we showed that on a global scale, aridity is an important regulator of the responses of total SOC and POC concentrations to drought, with mesic regions being susceptible to SOC losses under drought conditions. Drought-induced soil C loss in these productive systems and mesic environments is expected to accelerate climate change. Aligned with our findings, Smith *et al.* (*42*) analyzed data from 100 grassland and shrubland sites, revealing a significant decline in ANPP, a fundamental ecosystem function, under drought conditions. Given the catastrophic shifts in local productivity and plant cover and richness due to extreme drought, our results reinforce the potentially strong consequences for essential ecosystem services across grasslands and shrublands worldwide. These findings also suggest that quantifying not only total SOC but also POC and MAOC will enable us to better estimate the vulnerability of soil C to climate change, understand the underlying mechanisms of changes in soil C storage, and improve predictive capacity of Earth system models as the frequency and duration of drought events increase under future global change scenarios.

## MATERIALS AND METHODS

### Study design and study sites

The 19 sites in this study are part of IDE (droughtnet.weebly.com) (*42*). These sites included two ecosystem types (13 grasslands and 6 shrublands), encompassing a wide range of climatic and soil conditions including mean annual temperature (−1.2° to 17.6°C), MAP (215 to 1012 mm) and SOC concentrations (6.1 to 71.9 g kg^−1^). (Fig. 1 and table S1). At each site, three replicate plots were sampled for each treatment (control and drought). The number of replicates for both control and drought treatments at each site was low, potentially limiting the statistical power to discern treatment effects at any specific site. However, IDE provided a distinctive opportunity to investigate generalized patterns in terms of grassland and shrubland soil C responses to drought enrichment across extensive geographical scales, as well as how environmental factors may mediate these responses (*n* = 114 plots). The IDE used fixed rainout shelters (each a minimum of 2 m by 2 m) to impose drought treatments by reducing each precipitation event (table S2). For the drought treatment, the percentage reduction of each rainfall event mimicked an extreme drought, defined as an extreme reduction in precipitation (based on the first percentile of the long-term record). That is, drought treatments were meant to mimic a 1-in-100-year drought, which would depend on each particular site. This is the principle of the IDE to attain a common target level of extremity. For this, the magnitude of treatment at each site (precipitation reduction) was determined using site-specific climate data spanning over 100 years or interpolated data spanning 100 years from the Terrestrial Precipitation Analysis tool (*42*, *43*). The percentage reduction in annual precipitation imposed at each site ranged from 32 to 70% (table S1). Sites were subjected to drought between 1 and 5 years (fig. S1). Some sites had undergone plowing, grazing, burning, or mowing practices within three decades before establishment of the experiment, but only three sites that we know of were actively mowed (*n* = 2) or burned (*n* = 1). Further details regarding disturbance and management history for each site can be found in table S2.

### Sample collection and analysis

ANPP was measured annually at the plot level using ecosystem-specific methods (*44*). Further details regarding the measurement and calculation of ANPP can be found in the study of Smith *et al.* (*42*). After 1 to 5 years of drought treatment, three soil samples at a depth of 0 to 15 cm were collected using an auger from each plot to capture spatial variability and composited into one sample per plot. Soils were sieved (2-mm mesh) to remove coarse residues and stones and air-dried. Soil texture was measured by a Mastersizer 2000 (Malvern Instruments Ltd., Southborough, MA, USA). Soil pH was measured in soil/deionized water (1:5, w/v) slurry using a PHS-3E glass pH electrode (Leichi Inc., Shanghai, China). The concentrations of total polyphenols were determined using the Folin-Ciocalteu method (*45*). WHC was determined by saturating 10 g of soil with deionized water over a filter paper (Whatman 2) placed in a funnel. Then, the soil was drained over 2 hours, and WHC was determined gravimetrically by drying soil for 48 hours at 105°C.

Amino sugars, including muramic acid (MurN), glucosamine (GlcN), and galactosamine (GalN), were used as biomarkers to determine soil fungal and bacterial necromass (*46*). Amino sugar concentration in soil samples was determined according to the method described by Zhang and Amelung (*47*). Briefly, 3 g of air-dried soil was hydrolyzed with 6 M HCl at 105°C for 8 hours. Then, myo-inositol was added to each sample as a recovery standard. The hydrolysates were filtered and dried at 52°C under reduced pressure on a rotary evaporator. After redissolving the hydrolysates in deionized water and adjusting the pH to 6.6 to 6.8 using 1 M KOH and 0.01 M HCl, the pH-adjusted samples were centrifuged for 10 min, and the supernatant was dried at 52°C under reduced pressure on a rotary evaporator. The residues were dissolved in methanol and separated from salts by centrifugation. Methyl-glucamine was added to each sample as a quantitative standard. After adding 300 μl of derivatization reagent, amino sugars were transformed into aldononitrile derivatives by heating at 75°C for 0.5 hours. The derivatives were further acetylated at 80°C for 20 min and then extracted using 1.5 ml of dichloromethane. Excessive derivatization reagents were removed with 1 M HCl and deionized water. The amino sugar derivatives were dried under N_2_ and redissolved in 300 μl of hexane and ethyl acetate mixture (1:1, v/v). The extracted amino sugars were quantified using a gas chromatograph (Agilent 6890, Agilent Technologies, Wilmington, DE, USA). The peaks of individual amino sugar derivatives were identified by comparing them with the retention time of standards. According to Zhang and Amelung (*47*), recoveries of amino sugars during hydrolysis were assessed by hydrolysis of amino sugars with or without soil in the presence of myo-inositol as internal standard. Recoveries of amino sugars in the purification procedure were estimated by standard addition. Fungal C was calculated by subtracting bacterial GlcN from total GlcN, assuming that MurN acid and GlcN occur at a ratio of 1 to 2 M in bacterial cells: millimole of fungal C per gram of dry weight = (millimole of GlcN − 2 × millimole of MurN) × 9 (*48*). Bacterial C was calculated by multiplying the concentration of MurN by 45 (*49*). Microbial necromass C was estimated as the sum of fungal and bacterial C.

We conducted an isotope-labeled glucose addition experiment with two substrate treatments: deionized water and ^13^C-labeled glucose, to elucidate the priming effect. Briefly, the experimental setup involved placing 20 g (dry weight) of soil into 150-ml plastic jars, totaling 228 jars, based on a design of 19 sites × 2 drought treatments × 3 replicates × 2 substrate addition treatments. Soil moisture was adjusted to 50% WHC using deionized water. Before treatment, all jars were preincubated at 25°C for 7 days to mitigate immediate pulse effects on microbial activity. Following preincubation, a glucose solution was administered to one of the groups using a pipette, providing 2000 μg of C g^−1^ of soil as ^13^C-labeled glucose (5 atomic %). The control group received an equivalent volume of deionized water. CO_2_ production in each jar was measured on days 1, 2, 3, 7, 15, 23, and 35 of incubation. The jars were placed in a 16-hole electric water bath set at 25°C, controlled by an automatic temperature regulator, and connected to a PICARRO isotope analyzer (G2131-i, PICARRO Inc., Sunnyvale, CA, USA), which recorded δ^13^CO_2_ and ^12^CO_2_ concentrations every second. Each soil sample underwent 120 measurements during the observation period. The proportions of CO_2_ derived from SOC (*R*_SOC_) in total CO_2_ release (*R*_S_) were calculated using a mass balance model based on Waldrop and Firestone (*50*)

$$R_{\text{SOC}}=R_{S}\times \left[1-\frac{\left(\delta_{\text{addition}}-\delta_{\text{control}}\right)}{\left(\delta_{\text{Glu}}-\delta_{\text{control}}\right)}\right]$$ (1)

where δ_addition_ and δ_control_ represent the δ^13^C values of CO_2_ from the glucose addition and control treatment soils, respectively, while δ_Glu_ is the δ^13^C value of the added glucose. The intensity of the priming effect induced by glucose addition was determined by comparing the difference in SOC-derived CO_2_-C between the glucose addition and control treatments over the 35-day incubation period.

All soil samples were fractionated to obtain POM and MAOM using a wet sieving approach described by Leuthold *et al.* (*51*). Briefly, 5 g of air-dried, 2-mm sieved soil were dispersed in a 0.5% sodium hexametaphosphate solution by shaking for 18 hours with glass beads on a reciprocating shaker at a low speed (112 oscillations/min). Dispersed samples were passed through 53-μm sieves and rinsed with deionized water to separate the POM (>53 μm) from the MAOM (<53 μm). All fractions were dried at 60°C to constant mass, weighed, and then analyzed for total C and nitrogen (TN) concentrations using an elemental analyzer (vario EL cube, Elementar, Germany). Soils were pretreated with dilute HCl to remove inorganic C. After fractionation, mass recoveries ranged from 94 to 104%, with an average of 100%, and C recoveries ranged from 86 to 114%, with an average of 95%.

Global AI information was downloaded from the CGIAR-CSI GeoPortal (https://cgiarcsi.community) (*52*). Long-term mean annual net primary productivity, precipitation, and potential evapotranspiration data were obtained from the high-resolution BIOCLIM datasets (*53*).

### Data and statistical analysis

We calculated the yearly rate of change in total SOC concentrations and in the two soil C fractions (POC and MAOC) between the drought and control treatments as follows

ΔDDTOC=(DTOC−CTOC)/yrs (2)

$$\text{R}\text{TOC}=\left[\text{CTOC}+\left(\text{DTOC}-\text{CTOC}\right)/\text{yrs}\right]/\text{CTOC}$$ (3)

where ΔDDTOC is the total change in total SOC concentration or its fractions (POC or MAOC) (in grams per kilogram per year), *R*TOC is the proportional changes in total SOC concentration or its fractions in response to drought, DTOC is the total SOC concentration or its fractions (in grams per kilogram) under the drought treatment, CTOC is total SOC concentration or its fractions (in grams per kilogram) under the control treatment, yrs is the number of years drought treatments were imposed before soil sample collection. The total change in priming effect (ΔDDPE) was determined by comparing the difference in the priming effect (in milligrams per kilogram) between the drought and control treatments.

The AI value (0.65) is important as it represents the transition between drylands and nondryland ecosystems (*32*). Thus, AI = 0.65 was used as the threshold for linear mixed-effects model analyses. To examine the effects of AI and its interaction with drought treatment on total SOC, POC, and MAOC concentrations, as well as environmental factors (ANPP, TN, SOC:TN, WHC, soil phenols, microbial necromass C, and soil pH), we carried out a series of linear mixed-effects models. These models included drought treatments, AI, and their interaction as fixed effects and sites as random effect. Linear mixed-effects models were performed of “lme4” package. We used bootstrapping [1000 simulations, function confint.merMod(method = “boot”)] to derive 95% confidence intervals for coefficients from linear mixed model. The relationships between the ΔDDTOC, RTOC, AI, standing SOC concentration, CV in precipitation, WHC, and ΔDDPE were examined using a second-order polynomial fit or linear regression. Graphs were generated using “ggplot2” package (*54*).

## Supplementary Materials

### This PDF file includes:

Figs. S1 to S4 Tables S1 and S2 References
