# Supplementary material for: Aridity drives the response of soil total and particulate organic carbon to drought in temperate grasslands and shrublands
Source: Sci Adv. 2024 Oct 4;10(40):eadq2654. doi: 10.1126/sciadv.adq2654 (PMC11451523; doi:10.1126/sciadv.adq2654)
Supplement: Supplementary file 1 — Figs. S1 to S4 Tables S1 and S2 References [file sciadv.adq2654_sm.pdf]

Science Advances
AAAS

# Supplementary Materials for

## Aridity drives the response of soil total and particulate organic carbon to drought in temperate grasslands and shrublands

Baoku Shi *et al.*

Corresponding author: Melinda D. Smith, melinda.smith@colostate.edu; Wei Sun, sunwei@nenu.edu.cn

*Sci. Adv.* **10**, eadq2654 (2024)
DOI: 10.1126/sciadv.adq2654

**This PDF file includes:**

Figs. S1 to S4
Tables S1 and S2
References

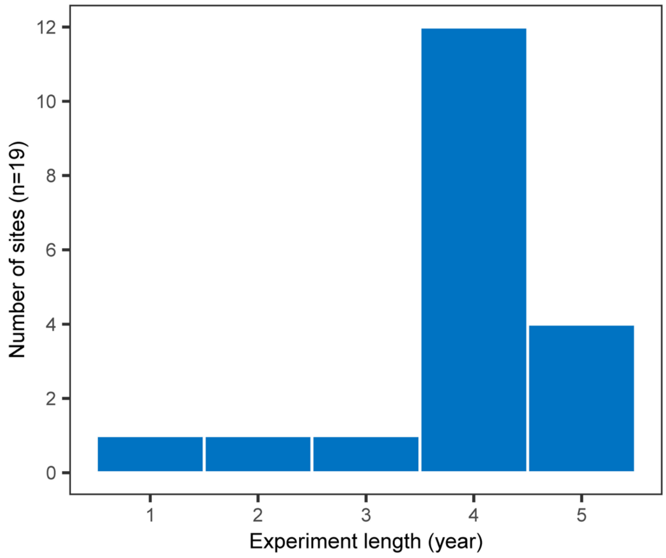


**Fig. S1. Frequency of sites by experiment length.**

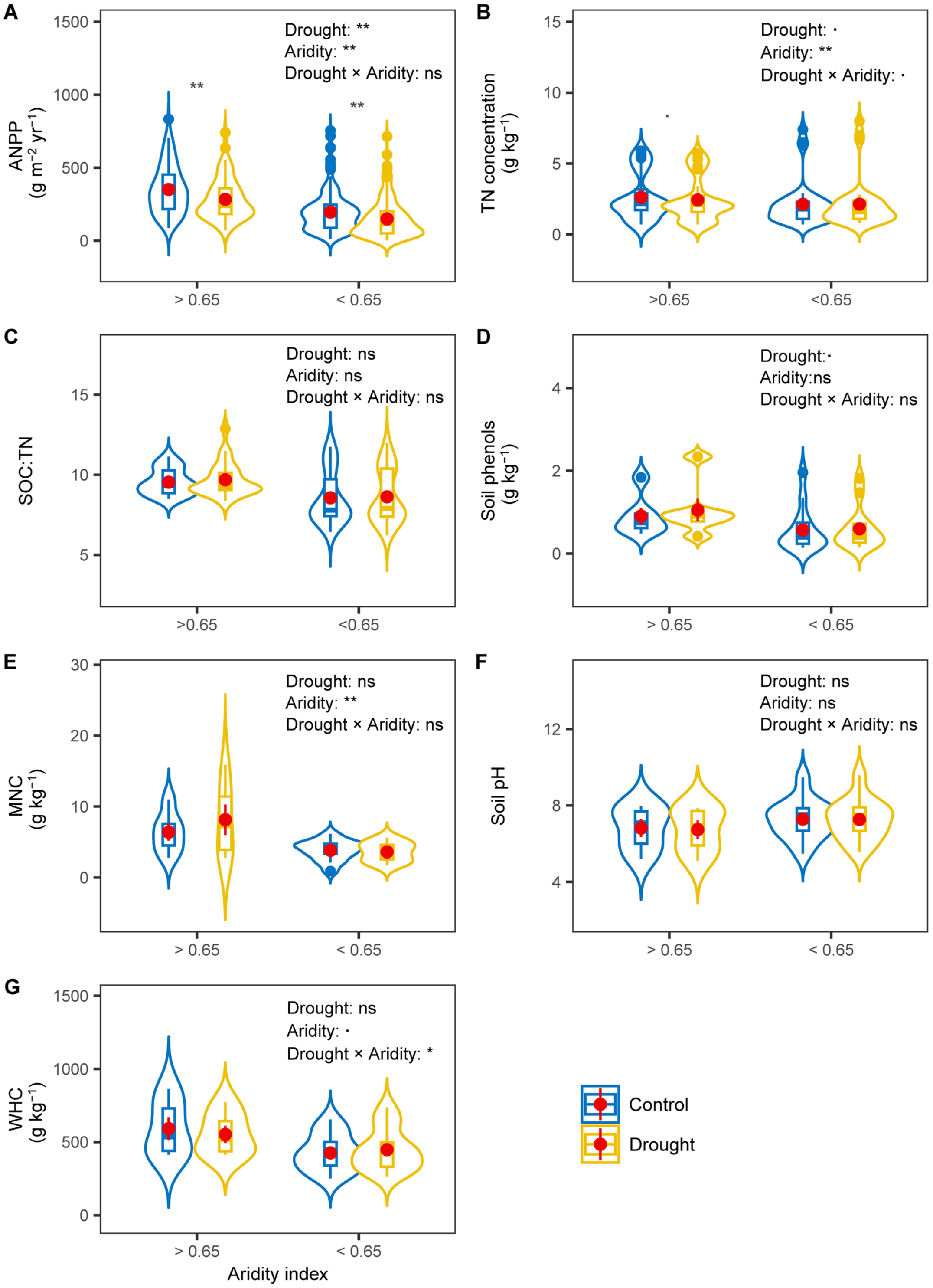


**Fig. S2. Aridity index-moderated responses of environmental factors to drought. The responses of (A) aboveground net primary productivity (ANPP), (B) total nitrogen (TN) concentration, (C) the ratio between soil organic carbon and TN (SOC: TN), (D) soil phenols, (E) microbial necromass C (MNC), (F) soil pH and (G) water holding capacity (WHC) to drought as moderated by the aridity index.** Red dot shows the mean. **$P < 0.01$; *$P < 0.05$; $\cdot P < 0.1$; ns: $P > 0.05$.

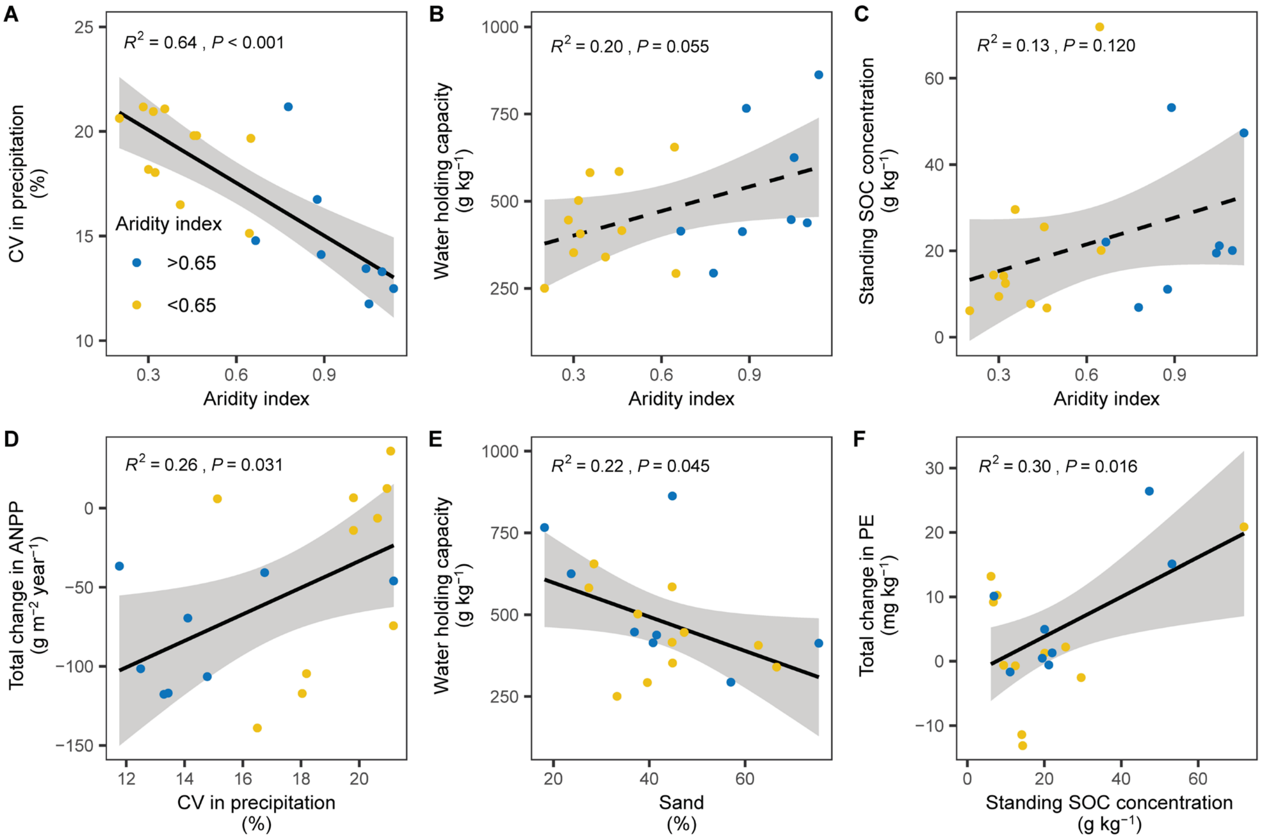


**Fig. S3. Interconnections between environmental factors, soil properties, and soil organic carbon across aridity gradients. Relationships between (A) coefficient of variation (CV) in precipitation and the aridity index, (B) water holding capacity with the aridity index, (C) standing soil organic carbon (SOC) concentration and the aridity index, (D) total change in aboveground net primary productivity (ANPP) and CV in precipitation, (E) water holding capacity and sand, and (F) total change in priming effect and standing SOC concentration.** Solid lines indicate significant correlations, while dashed lines indicate non-significant correlations. Shaded areas reflect the 95% CI for the line of fit.

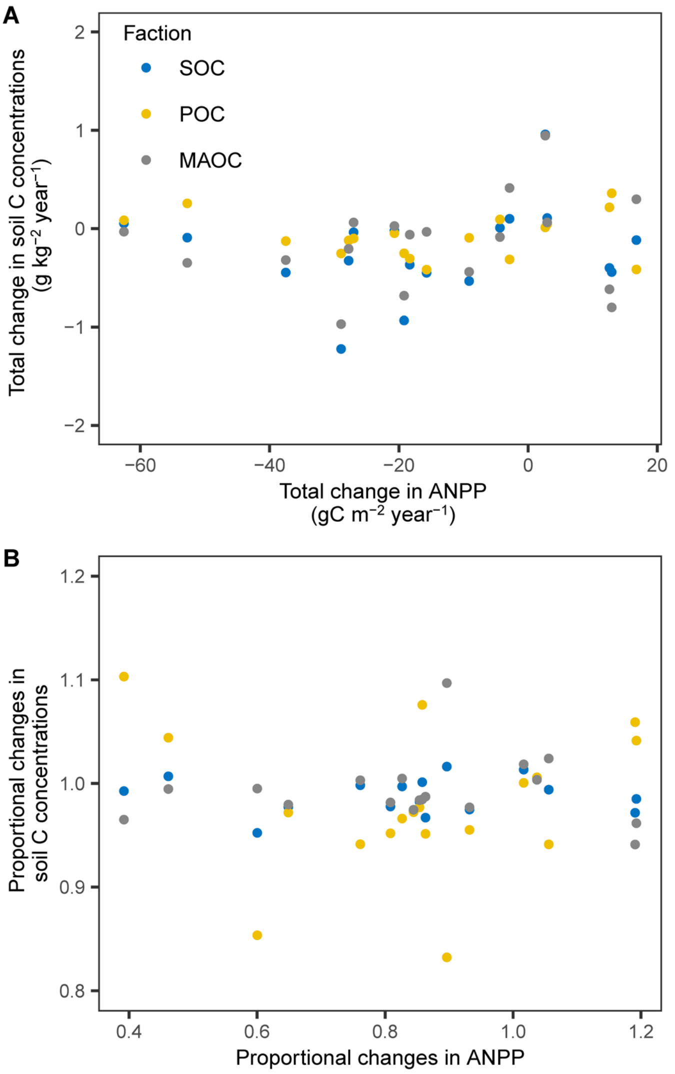


**Fig. S4. Changes in soil carbon in relation to changes in aboveground net primary productivity (ANPP). Relationships between (A) the total changes and (B) proportional changes in soil organic carbon (SOC), particulate organic carbon (POC), and mineral-associated organic carbon (MAOC) concentrations with the change in ANPP.** ANPP mass production was converted to carbon by multiplying by a conversion factor of 0.45 (*55*).

**Table S1. Environmental characteristics of the 19 International Drought Experiment (IDE) study sites.** Site codes: combine the site names and two-letter country codes. MAT: mean annual temperature; MAP: mean annual precipitation. Some of the data comes from Smith et al. (*42*).

| Site code | Ecosystem type | Continent | Latitude (°) | Longitude (°) | MAP (mm) | MAT (°C) | Aridity index | Soil pH | Sand content (%) | Drought duration (year) | Precipitation reduction (%) |
|---|---|---|---|---|---|---|---|---|---|---|---|
| baddrt.de | Grassland | Europe | 51.3921 | 11.8780 | 489 | 9.5 | 0.67 | 7.02 | 40.79 | 4.5 | 55 |
| bayrdrt.de | Grassland | Europe | 49.9220 | 11.5838 | 724 | 8.3 | 1.10 | 5.21 | 41.53 | 5 | 40 |
| brookdale.ca | Grassland | North America | 50.0510 | -99.9200 | 474 | 2.2 | 0.64 | 7.56 | 28.45 | 4 | 32 |
| cedarsav.us | Grassland | North America | 45.3972 | -93.1803 | 682 | 7.5 | 0.88 | 6.50 | 75.37 | 3 | 43 |
| chang.cn | Grassland | Asia | 44.7500 | 123.7500 | 445 | 5.6 | 0.41 | 9.47 | 66.57 | 5 | 70 |
| ciempoz.es | Shrubland | Europe | 40.1899 | -3.6014 | 364 | 15.2 | 0.32 | 7.52 | 37.60 | 4 | 44 |
| esw.ca | Grassland | North America | 43.0800 | -81.3400 | 1012 | 7.9 | 1.05 | 7.96 | 23.69 | 4 | 45 |
| hard.us | Shrubland | North America | 41.6147 | -111.5669 | 462 | 9.0 | 0.36 | 7.86 | 27.38 | 4 | 47 |
| jenadrt.de | Grassland | Europe | 50.9378 | 11.5304 | 587 | 9.5 | 0.89 | 7.70 | 18.14 | 4.5 | 66 |
| maodeng.cn | Grassland | Asia | 44.1000 | 116.2800 | 215 | -1.2 | 0.32 | 8.35 | 62.75 | 4 | 50 |
| octc.us | Shrubland | North America | 43.2548 | -116.2174 | 269 | 10.3 | 0.20 | 7.57 | 33.27 | 1 | 40 |
| oreaa.us | Shrubland | North America | 42.1840 | -121.0150 | 497 | 7.1 | 0.46 | 6.72 | 44.76 | 4 | 41 |
| oreac.us | Shrubland | North America | 42.1830 | -121.0150 | 497 | 7.1 | 0.46 | 6.54 | 44.76 | 4 | 41 |
| rhijn.nl | Grassland | Europe | 52.0735 | 5.1757 | 797 | 9.5 | 1.04 | 7.66 | 36.91 | 4 | 51 |
| sgsdrt.us | Grassland | North America | 40.8053 | -104.7147 | 344 | 8.4 | 0.30 | 6.67 | 44.86 | 2 | 66 |
| siziwang.cn | Grassland | Asia | 41.8000 | 111.9000 | 220 | 3.5 | 0.28 | 7.86 | 47.28 | 4.5 | 50 |
| taihang.cn | Shrubland | Asia | 37.5244 | 114.1550 | 560 | 13 | 0.65 | 6.05 | 39.61 | 5 | 60 |
| validate.fr | Grassland | Europe | 45.7222 | 3.0222 | 790 | 8.19 | 1.14 | 5.84 | 44.82 | 4.5 | 68 |
| yarradrt.au | Grassland | Australia | -33.6136 | 150.7381 | 801 | 17.6 | 0.78 | 5.47 | 57.02 | 5 | 65 |

**Table S2. Disturbance and management history and the size of rainout shelters for each site.** The "plowing history" indicates mechanical disruptions that involve the breaking up of the soil surface. The "fire history" indicates disturbance events caused by both natural wildfires and intentionally ignited, controlled burns. The "grazing history" indicates the presence of both wild and domesticated grazing animals in the area. The "mowing history" indicates the removal of aboveground biomass using mechanical methods. Y indicates a disturbance or management practice had generally occurred within the three decades prior to the establishment of the experiment, N indicates there were no such disturbances or management practices and *Indicates that the activity took place while the experiment was ongoing. NA indicates no available information. Some of the data comes from Smith et al. (*42*).

| Site code | Plowing history | Grazing history | Burning history | Mowing history | Shelter (length × width × hgt, m) |
|---|---|---|---|---|---|
| baddrt.de | N | N | N | Y* | 3 × 3 × 2 |
| bayrdrt.de | N | N | N | Y* | 8 × 6 × 2.5 |
| brookdale.ca | N | N | N | N | 2.5 × 2.5 × 0.8 |
| cedarsav.us | N | N | Y* | N | NA |
| chang.cn | N | N | N | N | 3.5 × 3.5 × 1.2 |
| ciempoz.es | N | N | N | N | 3 × 3 × 1.9 |
| esw.ca | Y | N | N | N | 3 × 3 × 1 |
| hard.us | N | Y | N | N | 3 × 3 × 2 |
| jenadrt.de | N | N | N | N | 3 × 3 × 2 |
| maodeng.cn | N | N | N | N | 3 × 3 × 2 |
| octc.us | N | Y | N | N | 5 × 2.5 × 1.75 |
| oreaa.us | N | N | N | N | 3 × 3 × 2 |
| oreac.us | N | N | N | N | 3 × 3 × 2 |
| rhijn.nl | N | Y | N | N | 3 × 3 × 1.3 |
| sgsdrt.us | N | N | N | N | NA |
| siziwang.cn | N | N | N | N | 3 × 3 × 2 |
| taihang.cn | N | N | N | N | 6 × 6 × 5 |
| validate.fr | N | N | N | Y | 3 × 3 × 1.4 |
| yarradrt.au | N | Y | N | N | 3 × 3 × 1.6 |

## REFERENCES AND NOTES


1. S. E. Trumbore, O. A. Chadwick, R. Amundson, Rapid exchange between soil carbon and atmospheric carbon dioxide driven by temperature change. *Science* **272**, 393–396 (1996).

2. T. W. Crowther, C. Riggs, E. M. Lind, E. T. Borer, E. W. Seabloom, S. E. Hobbie, J. Wubs, P. B. Adler, J. Firn, L. Gherardi, N. Hagenah, K. S. Hofmockel, J. M. H. Knops, R. L. McCulley, A. S. MacDougall, P. L. Peri, S. M. Prober, C. J. Stevens, D. Routh, Sensitivity of global soil carbon stocks to combined nutrient enrichment. *Ecol. Lett.* **22**, 936–945 (2019).

3. World Meteorological Organization (WMO), "Report on drought and countries affected by drought during 1974-1985" (WMO/TD-No. 133, WMO, 1986); https://library.wmo.int/idurl/4/44107.

4. I. J. Slette, A. K. Post, M. Awad, T. Even, A. Punzalan, S. Williams, M. D. Smith, A. K. Knapp, How ecologists define drought, and why we should do better. *Glob. Chang. Biol.* **25**, 3193–3200 (2019).

5. M. K. van der Molen, A. J. Dolman, P. Ciais, T. Eglin, N. Gobron, B. E. Law, P. Meir, W. Peters, O. L. Phillips, M. Reichstein, T. Chen, S. C. Dekker, M. Doubková, M. A. Friedl, M. Jung, B. J. J. M. van den Hurk, R. A. M. de Jeu, B. Kruijt, T. Ohta, K. T. Rebel, S. Plummer, S. I. Seneviratne, S. Sitch, A. J. Teuling, G. R. van der Werf, G. Wang, Drought and ecosystem carbon cycling. *Agric. For. Meteorol.* **151**, 765–773 (2011).

6. A. Dai, Increasing drought under global warming in observations and models. *Nat. Clim. Change* **3**, 52–58 (2013).

7. H.-O. Pörtner, D. C. Roberts, H. Adams, C. Adler, P. Aldunce, E. Ali, R. A. Begum, R. Betts, R. B. Kerr, R. Biesbroek, others, *Climate Change 2022: Impacts, Adaptation and Vulnerability* (IPCC, 2022).

8. M. Zhao, S. W. Running, Drought-induced reduction in global terrestrial net primary production from 2000 through 2009. *Science* **329**, 940–943 (2010).

9. A. J. Felton, A. K. Knapp, M. D. Smith, Precipitation-productivity relationships and the duration of precipitation anomalies: An underappreciated dimension of climate change. *Glob. Change Biol.* **27**, 1127–1140 (2021).

10. A. Canarini, P. Mariotte, L. Ingram, A. Merchant, F. A. Dijkstra, Mineral-associated soil carbon is resistant to drought but sensitive to legumes and microbial biomass in an Australian grassland. *Ecosystems* **21**, 349–359 (2018).

11. X. Zhou, L. Zhou, Y. Nie, Y. Fu, Z. Du, J. Shao, Z. Zheng, X. Wang, Similar responses of soil carbon storage to drought and irrigation in terrestrial ecosystems but with contrasting mechanisms: A meta-analysis. *Agric. Ecosyst. Environ.* **228**, 70–81 (2016).

12. Y. Zheng, Y. Jin, R. Ma, D. Kong, X. Zhu-Barker, W. R. Horwath, S. Niu, H. Wang, X. Xiao, S. Liu, J. Zou, Drought shrinks terrestrial upland resilience to climate change. *Glob. Ecol. Biogeogr.* **29**, 1840–1851 (2020).

13. L. Deng, C. Peng, D.-G. Kim, J. Li, Y. Liu, X. Hai, Q. Liu, C. Huang, Z. Shangguan, Y. Kuzyakov, Drought effects on soil carbon and nitrogen dynamics in global natural ecosystems. *Earth Sci. Rev.* **214**, 103501 (2021).

14. C. T. Garten, A. T. Classen, R. J. Norby, Soil moisture surpasses elevated $CO_2$ and temperature as a control on soil carbon dynamics in a multi-factor climate change experiment. *Plant and Soil* **319**, 85–94 (2009).

15. S. M. Schaeffer, P. M. Homyak, C. M. Boot, D. Roux-Michollet, J. P. Schimel, Soil carbon and nitrogen dynamics throughout the summer drought in a California annual grassland. *Soil Biol. Biochem.* **115**, 54–62 (2017).

16. M. F. Cotrufo, M. G. Ranalli, M. L. Haddix, J. Six, E. Lugato, Soil carbon storage informed by particulate and mineral-associated organic matter. *Nat. Geosci.* **12**, 989–994 (2019).

17. K. S. Rocci, J. M. Lavallee, C. E. Stewart, M. F. Cotrufo, Soil organic carbon response to global environmental change depends on its distribution between mineral-associated and particulate organic matter: A meta-analysis. *Sci. Total Environ.* **793**, 148569 (2021).

18. A. B. Keller, E. T. Borer, S. L. Collins, L. C. DeLancey, P. A. Fay, K. S. Hofmockel, A. D. Leakey, M. A. Mayes, E. W. Seabloom, C. A. Walter, Y. Wang, Q. Zhao, S. E. Hobbie, Soil carbon stocks in temperate grasslands differ strongly across sites but are insensitive to decade-long fertilization. *Glob. Change Biol.* **28**, 1659–1677 (2022).

19. P. Sollins, P. Homann, B. A. Caldwell, Stabilization and destabilization of soil organic matter: Mechanisms and controls. *Geoderma* **74**, 65–105 (1996).

20. M. F. Cotrufo, J. M. Lavallee, Y. Zhang, P. M. Hansen, K. H. Paustian, M. Schipanski, M. D. Wallenstein, In-N-Out: A hierarchical framework to understand and predict soil carbon storage and nitrogen recycling. *Glob. Change Biol.* **27**, 4465–4468 (2021).

21. M. Kleber, K. Eusterhues, M. Keiluweit, C. Mikutta, R. Mikutta, P. S. Nico, Mineral-organic associations: Formation, properties, and relevance in soil environments. *Adv. Agron.* **130**, 1–140 (2015).

22. C. Liang, J. P. Schimel, J. D. Jastrow, The importance of anabolism in microbial control over soil carbon storage. *Nat. Microbiol.* **2**, 17105 (2017).

23. Y. Rui, R. D. Jackson, M. F. Cotrufo, G. R. Sanford, B. J. Spiesman, L. Deiss, S. W. Culman, C. Liang, M. D. Ruark, Persistent soil carbon enhanced in Mollisols by well-managed grasslands but not annual grain or dairy forage cropping systems. *Proc. Natl. Acad. Sci. U.S.A.* **119**, e2118931119 (2022).

24. M. F. Cotrufo, J. M. Lavallee, "Soil organic matter formation, persistence, and functioning: A synthesis of current understanding to inform its conservation and regeneration" in *Advances in Agronomy* (Elsevier, 2022), pp. 1–66; https://linkinghub.elsevier.com/retrieve/pii/S0065211321001048.

25. D. K. Benbi, A. K. Boparai, K. Brar, Decomposition of particulate organic matter is more sensitive to temperature than the mineral associated organic matter. *Soil Biol. Biochem.* **70**, 183–192 (2014).

26. A. Canarini, L. P. Kiær, F. A. Dijkstra, Soil carbon loss regulated by drought intensity and available substrate: A meta-analysis. *Soil Biol. Biochem.* **112**, 90–99 (2017).

27. R. R. Weil, N. C. Brady, *The Nature and Properties of Soils* (Pearson Press, 2017).

28. T. E. Huxman, M. D. Smith, P. A. Fay, A. K. Knapp, M. R. Shaw, M. E. Loik, S. D. Smith, D. T. Tissue, J. C. Zak, J. F. Weltzin, W. T. Pockman, O. E. Sala, B. M. Haddad, J. Harte, G. W. Koch, S. Schwinning, E. E. Small, D. G. Williams, Convergence across biomes to a common rain-use efficiency. *Nature* **429**, 651–654 (2004).

29. A. K. Knapp, C. Beier, D. D. Briske, A. T. Classen, Y. Luo, M. Reichstein, M. D. Smith, S. D. Smith, J. E. Bell, P. A. Fay, J. L. Heisler, S. W. Leavitt, R. Sherry, B. Smith, E. Weng, Consequences of more extreme precipitation regimes for terrestrial ecosystems. *Bioscience* **58**, 811–821 (2008).

30. L. H. Fraser, H. A. Henry, C. N. Carlyle, S. R. White, C. Beierkuhnlein, J. F. Cahill, B. B. Casper, E. Cleland, S. L. Collins, J. S. Dukes, A. K. Knapp, E. Lind, R. Long, Y. Luo, P. B. Reich, M. D. Smith, M. Sternberg, R. Turkington, Coordinated distributed experiments: An emerging tool for testing global hypotheses in ecology and environmental science. *Front. Ecol. Environ.* **11**, 147–155 (2013).

31. D. L. Hoover, K. R. Wilcox, K. E. Young, Experimental droughts with rainout shelters: A methodological review. *Ecosphere* **9**, e02088 (2018).

32. E. Arnold, *World Atlas of Desertification* (UNEP, 1992).

33. J. M. Lavallee, J. L. Soong, M. F. Cotrufo, Conceptualizing soil organic matter into particulate and mineral-associated forms to address global change in the 21st century. *Glob. Change Biol.* **26**, 261–273 (2020).

34. I. Kantola, M. Masters, E. DeLucia, Soil particulate organic matter increases under perennial bioenergy crop agriculture. *Soil Biol. Biochem.* **113**, 184–191 (2017).

35. S. H. Villarino, P. Pinto, R. B. Jackson, G. Piñeiro, Plant rhizodeposition: A key factor for soil organic matter formation in stable fractions. *Sci. Adv.* **7**, eabd3176 (2021).

36. M. Singh, B. Sarkar, B. Biswas, N. S. Bolan, G. J. Churchman, Relationship between soil clay mineralogy and carbon protection capacity as influenced by temperature and moisture. *Soil Biol. Biochem.* **109**, 95–106 (2017).

37. P. He, L.-J. Li, S.-S. Dai, X.-L. Guo, M. Nie, X. Yang, Y. Kuzyakov, Straw addition and low soil moisture decreased temperature sensitivity and activation energy of soil organic matter. *Geoderma* **442**, 116802 (2024).

38. M. Berdugo, M. Delgado-Baquerizo, S. Soliveres, R. Hernández-Clemente, Y. Zhao, J. J. Gaitán, N. Gross, H. Saiz, V. Maire, A. Lehmann, M. C. Rillig, R. V. Solé, F. T. Maestre, Global ecosystem thresholds driven by aridity. *Science* **367**, 787–790 (2020).

39. P. García-Palacios, N. Gross, J. Gaitán, F. T. Maestre, Climate mediates the biodiversity-ecosystem stability relationship globally. *Proc. Natl. Acad. Sci. U.S.A.* **115**, 8400–8405 (2018).

40. W. Hu, J. Ran, L. Dong, Q. Du, M. Ji, S. Yao, Y. Sun, C. Gong, Q. Hou, H. Gong, R. Chen, J. Lu, S. Xie, Z. Wang, H. Huang, X. Li, J. Xiong, R. Xia, M. Wei, D. Zhao, Y. Zhang, J. Li, H. Yang, X. Wang, Y. Deng, Y. Sun, H. Li, L. Zhang, Q. Chu, X. Li, M. Aqeel, A. Manan, M. A. Akram, X. Liu, R. Li, F. Li, C. Hou, J. Liu, J.-S. He, L. An, R. D. Bardgett, B. Schmid, J. Deng, Aridity-driven shift in biodiversity-soil multifunctionality relationships. *Nat. Commun.* **12**, 5350 (2021).

41. G. Kröel-Dulay, A. Mojzes, K. Szitár, M. Bahn, P. Batáry, C. Beier, M. Bilton, H. J. De Boeck, J. S. Dukes, M. Estiarte, P. Holub, A. Jentsch, I. K. Schmidt, J. Kreyling, S. Reinsch, K. S. Larsen, M. Sternberg, K. Tielbörger, A. Tietema, S. Vicca, J. Peñuelas, Field experiments underestimate aboveground biomass response to drought. *Nat. Ecol. Evol.* **6**, 540–545 (2022).

42. M. D. Smith, K. D. Wilkins, M. C. Holdrege, P. Wilfahrt, S. L. Collins, A. K. Knapp, O. E. Sala, J. S. Dukes, R. P. Phillips, L. Yahdjian, L. A. Gherardi, T. Ohlert, C. Beier, L. H. Fraser, A. Jentsch, M. E. Loik, F. T. Maestre, S. A. Power, Q. Yu, A. J. Felton, S. M. Munson, Y. Luo, H. Abdoli, M. Abedi, C. L. Alados, J. Alberti, M. Alon, H. An, B. Anacker, M. Anderson, H. Auge, S. Bachle, K. Bahalkeh, M. Bahn, A. Batbaatar, T. Bauerle, K. H. Beard,

K. Behn, I. Beil, L. Biancari, I. Blindow, V. F. Bondaruk, E. T. Borer, E. W. Bork, C. M. Bruschetti, K. M. Byrne, J. F. Cahill Jr., D. A. Calvo, M. Carbognani, A. Cardoni, C. N. Carlyle, M. Castillo-Garcia, S. X. Chang, J. Chieppa, M. V. Cianciaruso, O. Cohen, A. L. Cordeiro, D. F. Cusack, S. Dahlke, P. Daleo, C. M. D'Antonio, L. H. Dietterich, T. S. Doherty, M. Dubbert, A. Ebeling, N. Eisenhauer, F. M. Fischer, T. G. W. Forte, T. Gebauer, B. Gozalo, A. C. Greenville, K. G. Guidoni-Martins, H. J. Hannusch, S. V. Haugum, Y. Hautier, M. Hefting, H. A. L. Henry, D. Hoss, J. Ingrisch, O. Iribarne, F. Isbell, Y. Johnson, S. Jordan, E. F. Kelly, K. Kimmel, J. Kreyling, G. Kröel-Dulay, A. Kröpfl, A. Kübert, A. Kulmatiski, E. G. Lamb, K. S. Larsen, J. Larson, J. Lawson, C. V. Leder, A. Linstädter, J. Liu, S. Liu, A. G. Lodge, G. Longo, A. Loydi, J. Luan, F. C. Lubbe, C. Macfarlane, K. Mackie-Haas, A. V. Malyshev, A. Maturano-Ruiz, T. Merchant, D. B. Metcalfe, A. S. Mori, E. Mudongo, G. S. Newman, U. N. Nielsen, D. Nimmo, Y. Niu, P. Nobre, R. C. O'Connor, R. Ogaya, G. R. Oñatibia, I. Orbán, B. Osborne, R. Otfinowski, M. Pärtel, J. Penuelas, P. L. Peri, G. Peter, A. Petraglia, C. Picon-Cochard, V. D. Pillar, J. M. Piñeiro-Guerra, L. W. Ploughe, R. M. Plowes, C. Portales-Reyes, S. M. Prober, Y. Pueyo, S. C. Reed, E. G. Ritchie, D. A. Rodríguez, W. E. Rogers, C. Roscher, A. M. Sánchez, B. A. Santos, M. C. Scarfó, E. W. Seabloom, B. Shi, L. Souza, A. Stampfli, R. J. Standish, M. Sternberg, W. Sun, M. Sünnemann, M. Tedder, P. Thorvaldsen, D. Tian, K. Tielbörger, A. Valdecantos, L. Van Den Brink, V. Vandvik, M. R. Vankoughnett, L. G. Velle, C. Wang, Y. Wang, G. M. Wardle, C. Werner, C. Wei, G. Wiehl, J. L. Williams, A. A. Wolf, M. Zeiter, F. Zhang, J. Zhu, N. Zong, X. Zuo, Extreme drought impacts have been underestimated in grasslands and shrublands globally. *Proc. Natl. Acad. Sci. U.S.A.* **121**, e2309881120 (2024).

43. N. P. Lemoine, J. Sheffield, J. S. Dukes, A. K. Knapp, M. D. Smith, Terrestrial Precipitation Analysis (TPA): A resource for characterizing long-term precipitation regimes and extremes. *Methods Ecol. Evol.* **7**, 1396–1401 (2016).

44. T. J. Fahey, A. K. Knapp, *Principles and Standards for Measuring Primary Production* (Oxford Univ. Press, 2007).

45. R. Baziramakenga, R. R. Simard, G. D. Leroux, Determination of organic acids in soil extracts by ion chromatography. *Soil Biol. Biochem.* **27**, 349–356 (1995).

46. J. Parsons, "Chemistry and distribution of amino sugars in soils and soil organisms" in *Soil Biochemistry* (CRC Press, 1981) pp. 197–227.

47. X. Zhang, W. Amelung, Gas chromatographic determination of muramic acid, glucosamine, mannosamine, and galactosamine in soils. *Soil Biol. Biochem.* **28**, 1201–1206 (1996).

48. B. Engelking, H. Flessa, R. G. Joergensen, Shifts in amino sugar and ergosterol contents after addition of sucrose and cellulose to soil. *Soil Biol. Biochem.* **39**, 2111–2118 (2007).

49. A. Appuhn, R. Joergensen, Microbial colonisation of roots as a function of plant species. *Soil Biol. Biochem.* **38**, 1040–1051 (2006).

50. M. P. Waldrop, M. K. Firestone, Altered utilization patterns of young and old soil C by microorganisms caused by temperature shifts and N additions. *Biogeochemistry* **67**, 235–248 (2004).

51. S. J. Leuthold, M. L. Haddix, J. Lavallee, M. F. Cotrufo, "Physical fractionation techniques" in *Reference Module in Earth Systems and Environmental Sciences* (Elsevier, 2023), pp. 68–80; https://linkinghub.elsevier.com/retrieve/pii/B9780128229743000677.

52. R. J. Zomer, J. Xu, A. Trabucco, Version 3 of the global aridity index and potential evapotranspiration database. *Sci. Data.* **9**, 409 (2022).

53. D. N. Karger, O. Conrad, J. Böhner, T. Kawohl, H. Kreft, R. W. Soria-Auza, N. E. Zimmermann, H. P. Linder, M. Kessler, Climatologies at high resolution for the earth's land surface areas. *Sci. Data* **4**, 170122 (2017).

54. H. Wickham, *Ggplot2: Elegant Graphics for Data Analysis* (Springer International Publishing, 2009); http://link.springer.com/10.1007/978-3-319-24277-4.

55. J. Fang, S. Piao, C. B. Field, Y. Pan, Q. Guo, L. Zhou, C. Peng, S. Tao, Increasing net primary production in China from 1982 to 1999. *Front. Ecol. Environ.* **1**, 293–297 (2003).
